# Diaquabis­[3-(hydroxy­imino)­butanoato]nickel(II)

**DOI:** 10.1107/S1600536810004605

**Published:** 2010-02-10

**Authors:** Nikolay M. Dudarenko, Valentina A. Kalibabchuk, Maria L. Malysheva, Turganbay S. Iskenderov, Elżbieta Gumienna-Kontecka

**Affiliations:** a Department of Chemistry, Kiev National Taras Shevchenko University, Volodymyrska str. 64, 01601 Kiev, Ukraine; b Department of General Chemistry, O.O. Bohomolets National Medical University, Shevchenko blvd. 13, 01601 Kiev, Ukraine; c Department of Chemistry, Karakalpakian University, Universitet Keshesi 1, 742012 Nukus, Uzbekistan; d Faculty of Chemistry, University of Wrocław, 14 F. Joliot-Curie str., 50-383 Wrocław, Poland

## Abstract

In the neutral, mononuclear title complex, [Ni(C_4_H_6_NO_3_)_2_(H_2_O)_2_], the Ni atom lies on a crystallographic inversion centre within a distorted octa­hedral N_2_O_4_ environment. Two *trans*-disposed anions of 3-hydroxy­imino­butanoic acid occupy four equatorial sites, coordinated by the deprotonated carboxyl­ate and protonated oxime groups and forming six-membered chelate rings, while the two axial positions are occupied by the water O atoms. The O atom of the oxime group forms an intra­molecular hydrogen bond with the coordinated carboxyl­ate O atom. The complex mol­ecules are linked into chains along *b* by hydrogen bonds between the water O atom and the carboxyl­ate O of a neighbouring mol­ecule. The chains are linked by further hydrogen bonds into a layer structure.

## Related literature

For the coordination chemistry of 2-hydroxy­imino­propanoic acid and its amide derivatives, see: Onindo *et al.* (1995 ▶); Duda *et al.* (1997 ▶); Moroz *et al.* (2008 ▶). For 2-hydroxy­imino­carboxylic acids as efficient metal chelators, see: Onindo *et al.* (1995 ▶); Sliva *et al.* (1997*a* ▶,*b* ▶); Gumienna-Kontecka *et al.* (2000 ▶). For the use of 2-hydroxy­imino­carboxylic acid derivatives as efficient ligands for the stabilization of high oxidation states of transitional metals, see: Fritsky *et al.* (1998 ▶, 2006 ▶). For the structures of hydroxy­imino­carboxylic acid derivatives, see: Onindo *et al.* (1995 ▶); Sliva *et al.* (1997*a* ▶,*b* ▶); Mokhir *et al.* (2002 ▶). For structures with monodentately coordinated carboxylic groups, see: Wörl *et al.* (2005*a* ▶,*b* ▶). For the synthesis, see: Khromov (1950 ▶).
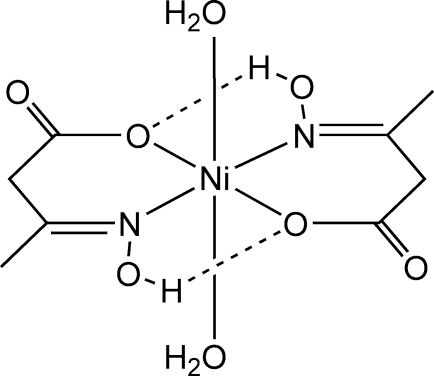


## Experimental

### 

#### Crystal data

[Ni(C_4_H_6_NO_3_)_2_(H_2_O)_2_]
*M* *_r_* = 326.94
Monoclinic, 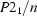

*a* = 9.6071 (9) Å
*b* = 7.1721 (7) Å
*c* = 9.6805 (9) Å
β = 107.557 (5)°
*V* = 635.94 (10) Å^3^
*Z* = 2
Mo *K*α radiation
μ = 1.56 mm^−1^
*T* = 120 K
0.23 × 0.15 × 0.11 mm

#### Data collection

Nonius KappaCCD diffractometer
Absorption correction: multi-scan (*SADABS*, Sheldrick, 2001 ▶) *T*_min_ = 0.622, *T*_max_ = 0.796
4576 measured reflections
1626 independent reflections
1286 reflections with *I* > 2σ(*I*)
*R*_int_ = 0.032

#### Refinement

*R*[*F* ^2^ > 2σ(*F* ^2^)] = 0.025
*wR*(*F* ^2^) = 0.060
*S* = 1.05
1626 reflections
101 parameters
H atoms treated by a mixture of independent and constrained refinement
Δρ_max_ = 0.35 e Å^−3^
Δρ_min_ = −0.32 e Å^−3^

### 

Data collection: *COLLECT* (Nonius, 2000 ▶); cell refinement: *DENZO*/*SCALEPACK* (Otwinowski & Minor, 1997 ▶); data reduction: *DENZO*/*SCALEPACK*; program(s) used to solve structure: *SIR2004* (Burla *et al.*, 2005 ▶); program(s) used to refine structure: *SHELXL97* (Sheldrick, 2008 ▶); molecular graphics: *ORTEP-3 for Windows* (Farrugia, 1997 ▶); software used to prepare material for publication: *SHELXL97*.

## Supplementary Material

Crystal structure: contains datablocks global, I. DOI: 10.1107/S1600536810004605/jh2130sup1.cif

Structure factors: contains datablocks I. DOI: 10.1107/S1600536810004605/jh2130Isup2.hkl

Additional supplementary materials:  crystallographic information; 3D view; checkCIF report

## Figures and Tables

**Table 1:** Hydrogen-bond geometry (Å, °)

*D*—H⋯*A*	*D*—H	H⋯*A*	*D*⋯*A*	*D*—H⋯*A*
O4—H2*O*4⋯O2^i^	0.79 (2)	1.94 (2)	2.7293 (17)	175 (2)
O3—H1*O*3⋯O1^ii^	0.72 (2)	2.10 (2)	2.7404 (17)	148 (2)
O4—H1*O*4⋯O2^iii^	0.87 (3)	1.90 (3)	2.7576 (16)	167 (2)

Symmetry codes: (i) 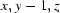; (ii) 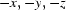; (iii) 
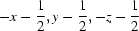
.

**Fig. 1.**
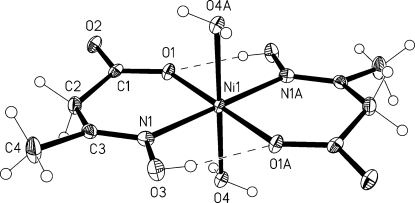
A view of compound (I), with displacement ellipsoids shown at the 50% probability level. H atoms are drawn as spheres of arbitrary radii. Hydrogen bonds are indicated by dashed lines. Symmetry code A: - x, - y, - z.

**Fig. 2.**
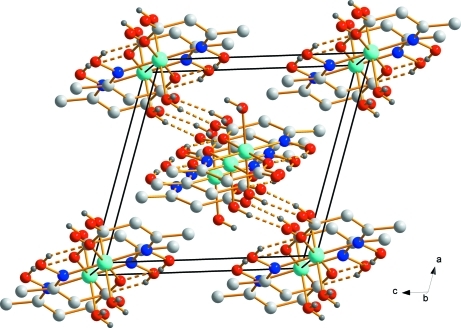
A packing diagram of the title compound. Hydrogen bonds are indicated by dashed lines. H atoms not involved in hydrogen bonding have been omitted for clarity.

## supplementary crystallographic information

### Comment

2-hydroxyiminopropanoic acid and its amide derivatives have been intensively studied during the past 15 years as efficient chelate ligands forming stable complexes with various transition metal ions (Onindo *et al.*, 1995; Duda *et al.*, 1997; Moroz *et al.*, 2008). The presence of an additional strong donor oxime function in the vicinity to the carboxylic group results in important increase of chelating efficiency as compare to structurally related amino acids. For example, 2-hydroxyiminopropanoic acid and other 2-hydroxyiminocarboxylic acids were shown to act as highly efficient chelators with respect to copper(II), nickel(II) and aluminium(III) (Onindo *et al.*, 1995; Sliva *et al.*, 1997a; Sliva *et al.*, 1997b; Gumienna-Kontecka *et al.*, 2000). Also, the amide derivatives of 2-hydroxyiminopropanoic acid possess strong σ-donor capacity and thus have been successfully used for preparation of metal complexes with efficient stabilization of Cu^3+^ and Ni^3+^ oxidation states (Fritsky *et al.*, 1998; Fritsky *et al.*, 2006). Surprisingly, that the complex formation properties of the nearest homologue of 2-hydroxyiminopropanoic acid - 2-hydroxyiminobutanoic acid - have not been studied at all up to date, and no crystal structures of the corresponding coordination compounds have been reported. Herein we present the first crystal structure of a metal complex of 3-hydroxyiminobutanoic acid.

A distorted octahedral coordination geometry is found in (I) with the Ni atom lying on a center of inversion, Fig. 1. Two four N atoms of two chelating oxime ligands define the equatorial plane, each defining a six-membered rings with a nearly planar conformation, and the two trans-coordinated water molecules complete the octahedral coordination geometry. The Ni-O bond lengths in the equatorial plane, Table 1, are somewhat shorter than the Ni-N (1.999 (1) Å and 2.043 (1) Å, respectively). The O atoms of the protonated oxime group form intramolecular hydrogen bonds with the coordinated carboxylate O atoms forming five-membered rings and thus fusing two six-membered chelate rings in a pseudomacrocyclic structure. The difference in C—O bond lengths for the coordinated and non-coordinated oxygen atoms (1.271 (2) Å and 1.250 (2)) Å is typical for monodentately coordinated carboxylic groups (Wörl *et al.*, 2005a,b). The C=N, C=O, N—O, bond lengths are typical for 2-hydroxyiminopropanoic acid and its derivatives (Onindo *et al.*, 1995; Sliva *et al.* (1997a,b); Mokhir *et al.*, 2002).

The octahedral complex molecules are organized in the chains disposed along b direction of the crystal due to H-bonds formed by the axial water molecules and non-coordinated carboxylate O atom O4 belonging to the translational molecule (Table 1). The chains are united in layers with the help of the H-bonds of different type (also formed by the water molecules and non-coordinated carboxylate O atom O4 belonging to another translational molecule). The layers disposed parallel to b direction of the crystal are united in three-dimensional structure only with the help of van der Waals contacts (Fig. 2).

### Experimental

Compound (I) was synthesized by adding the solution of nickel(II) nitrate hexahydrate (0.1 mmol, 0.029 g) in water (5 ml) to a solution of 3-hydroxyiminobutanoic acid (0.2 mmol, 0.023 g) in water (5 ml) with consequent heating at 60°C boiling over 15 min. The resultant solution was filtered and the dark pink filtrate was left to stand at room temperature. Slow evaporation of the solvent yielded lilac filtrate of (I) Yield 73%. 3-hydroxyiminobutanoic acid was prepared according to the reported procedure (Khromov, 1950).

### Refinement

The O—H hydrogen atoms were located from the difference Fourier map, and their coordinates and isotropic thermal parameters refined freely. The hydrogen atoms of the methyl and methylene groups were positioned geometrically and were constrained to ride on their parent atoms, with C—H = 0.96 Å, and *U*_iso_ = 1.5 *U*_eq_(parent atom) for the methyl groups, and with C—H = 0.97 Å, and *U*_iso_ = 1.2 *U*_eq_(parent atom) for the methylene groups.

### Crystal data

**Table:**

[Ni(C_4_H_6_NO_3_)_2_(H_2_O)_2_]	*F*(000) = 340
*M**_r_* = 326.94	*D*_x_ = 1.707 Mg m^−^^3^
Monoclinic, *P*2_1_/*n*	Mo *K*α radiation, λ = 0.71073 Å
Hall symbol: -P 2yn	Cell parameters from 3254 reflections
*a* = 9.6071 (9) Å	θ = 3.6–27.5°
*b* = 7.1721 (7) Å	µ = 1.56 mm^−^^1^
*c* = 9.6805 (9) Å	*T* = 120 K
β = 107.557 (5)°	Block, lilac
*V* = 635.94 (10) Å^3^	0.23 × 0.15 × 0.11 mm
*Z* = 2	

### Data collection

**Table:**

Nonius KappaCCD diffractometer	1626 independent reflections
Radiation source: fine-focus sealed tube	1286 reflections with *I* > 2σ(*I*)
horizontally mounted graphite crystal	*R*_int_ = 0.032
Detector resolution: 9 pixels mm^-1^	θ_max_ = 36.4°, θ_min_ = 3.6°
φ scans and ω scans with κ offset	*h* = −16→16
Absorption correction: multi-scan (*SADABS*, Sheldrick, 2001)	*k* = −11→11
*T*_min_ = 0.622, *T*_max_ = 0.796	*l* = −16→16
4576 measured reflections	

### Refinement

**Table:**

Refinement on *F*^2^	Primary atom site location: structure-invariant direct methods
Least-squares matrix: full	Secondary atom site location: difference Fourier map
*R*[*F*^2^ > 2σ(*F*^2^)] = 0.025	Hydrogen site location: inferred from neighbouring sites
*wR*(*F*^2^) = 0.060	H atoms treated by a mixture of independent and constrained refinement
*S* = 1.05	*w* = 1/[σ^2^(*F*_o_^2^) + (0.0307*P*)^2^] where *P* = (*F*_o_^2^ + 2*F*_c_^2^)/3
1626 reflections	(Δ/σ)_max_ < 0.001
101 parameters	Δρ_max_ = 0.35 e Å^−^^3^
0 restraints	Δρ_min_ = −0.32 e Å^−^^3^

### Special details

**Table:**

Geometry. All esds (except the esd in the dihedral angle between two l.s. planes) are estimated using the full covariance matrix. The cell esds are taken into account individually in the estimation of esds in distances, angles and torsion angles; correlations between esds in cell parameters are only used when they are defined by crystal symmetry. An approximate (isotropic) treatment of cell esds is used for estimating esds involving l.s. planes.
Refinement. Refinement of F^2^ against ALL reflections. The weighted R-factor wR and goodness of fit S are based on F^2^, conventional R-factors R are based on F, with F set to zero for negative F^2^. The threshold expression of F^2^ > 2sigma(F^2^) is used only for calculating R-factors(gt) etc. and is not relevant to the choice of reflections for refinement. R-factors based on F^2^ are statistically about twice as large as those based on F, and R- factors based on ALL data will be even larger.

### Fractional atomic coordinates and isotropic or equivalent isotropic displacement parameters (Å^2^)

**Table:**

	*x*	*y*	*z*	*U*_iso_*/*U*_eq_	
Ni1	0.0000	0.0000	0.0000	0.00914 (9)	
O1	−0.07925 (12)	0.23226 (15)	−0.10969 (11)	0.0129 (2)	
O2	−0.16372 (12)	0.51946 (16)	−0.14813 (12)	0.0144 (3)	
O3	0.00013 (14)	−0.01018 (19)	0.30386 (13)	0.0163 (3)	
O4	−0.20766 (13)	−0.12137 (18)	−0.07790 (13)	0.0129 (3)	
N1	−0.05011 (15)	0.09879 (19)	0.17719 (13)	0.0114 (3)	
C1	−0.13309 (17)	0.3773 (2)	−0.07010 (16)	0.0110 (3)	
C2	−0.1685 (2)	0.3862 (2)	0.07312 (17)	0.0150 (3)	
H2A	−0.1448	0.5116	0.1102	0.018*	
H2B	−0.2736	0.3737	0.0495	0.018*	
C3	−0.10320 (18)	0.2559 (2)	0.19819 (17)	0.0122 (3)	
C4	−0.1086 (2)	0.3230 (3)	0.34280 (18)	0.0226 (4)	
H4A	−0.0824	0.2229	0.4116	0.034*	
H4B	−0.2056	0.3649	0.3350	0.034*	
H4C	−0.0412	0.4243	0.3746	0.034*	
H1O3	0.038 (3)	−0.085 (3)	0.281 (2)	0.026 (7)*	
H1O4	−0.253 (3)	−0.063 (3)	−0.158 (3)	0.036 (6)*	
H2O4	−0.201 (2)	−0.227 (3)	−0.099 (2)	0.028 (6)*	

### Atomic displacement parameters (Å^2^)

**Table:**

	*U*^11^	*U*^22^	*U*^33^	*U*^12^	*U*^13^	*U*^23^
Ni1	0.01179 (16)	0.00714 (14)	0.00827 (13)	0.00080 (13)	0.00266 (10)	−0.00010 (12)
O1	0.0177 (7)	0.0086 (6)	0.0119 (5)	0.0016 (5)	0.0037 (5)	0.0000 (4)
O2	0.0171 (6)	0.0095 (6)	0.0139 (5)	0.0014 (5)	0.0004 (5)	0.0008 (4)
O3	0.0226 (7)	0.0163 (6)	0.0109 (5)	0.0079 (6)	0.0066 (5)	0.0046 (5)
O4	0.0156 (7)	0.0096 (6)	0.0126 (6)	0.0009 (5)	0.0029 (5)	−0.0003 (5)
N1	0.0119 (7)	0.0131 (7)	0.0085 (6)	0.0000 (6)	0.0021 (5)	0.0022 (5)
C1	0.0082 (8)	0.0095 (8)	0.0120 (7)	−0.0021 (6)	−0.0021 (6)	−0.0014 (6)
C2	0.0173 (9)	0.0119 (8)	0.0167 (8)	0.0029 (7)	0.0064 (7)	−0.0011 (6)
C3	0.0104 (8)	0.0138 (8)	0.0130 (7)	−0.0012 (7)	0.0042 (6)	−0.0016 (6)
C4	0.0324 (12)	0.0199 (10)	0.0177 (9)	0.0072 (8)	0.0110 (8)	−0.0031 (7)

### Geometric parameters (Å, °)

**Table:**

Ni1—O1^i^	1.9986 (10)	O4—H2O4	0.79 (2)
Ni1—O1	1.9986 (10)	N1—C3	1.278 (2)
Ni1—N1	2.0431 (13)	C1—C2	1.525 (2)
Ni1—N1^i^	2.0431 (13)	C2—C3	1.508 (2)
Ni1—O4^i^	2.0973 (12)	C2—H2A	0.9700
Ni1—O4	2.0973 (12)	C2—H2B	0.9700
O1—C1	1.2714 (18)	C3—C4	1.496 (2)
O2—C1	1.2499 (19)	C4—H4A	0.9600
O3—N1	1.4108 (17)	C4—H4B	0.9600
O3—H1O3	0.72 (2)	C4—H4C	0.9600
O4—H1O4	0.87 (3)		
			
O1^i^—Ni1—O1	180.00 (7)	C3—N1—Ni1	130.22 (11)
O1^i^—Ni1—N1	89.51 (5)	O3—N1—Ni1	115.60 (10)
O1—Ni1—N1	90.49 (5)	O2—C1—O1	121.88 (14)
O1^i^—Ni1—N1^i^	90.49 (5)	O2—C1—C2	116.05 (14)
O1—Ni1—N1^i^	89.51 (5)	O1—C1—C2	122.04 (14)
N1—Ni1—N1^i^	180.00 (7)	C3—C2—C1	123.47 (14)
O1^i^—Ni1—O4^i^	89.21 (5)	C3—C2—H2A	106.5
O1—Ni1—O4^i^	90.79 (5)	C1—C2—H2A	106.5
N1—Ni1—O4^i^	89.63 (5)	C3—C2—H2B	106.5
N1^i^—Ni1—O4^i^	90.37 (5)	C1—C2—H2B	106.5
O1^i^—Ni1—O4	90.79 (5)	H2A—C2—H2B	106.5
O1—Ni1—O4	89.21 (5)	N1—C3—C4	124.10 (15)
N1—Ni1—O4	90.37 (5)	N1—C3—C2	120.51 (14)
N1^i^—Ni1—O4	89.63 (5)	C4—C3—C2	115.38 (14)
O4^i^—Ni1—O4	180.00 (4)	C3—C4—H4A	109.5
C1—O1—Ni1	130.26 (10)	C3—C4—H4B	109.5
N1—O3—H1O3	102.5 (18)	H4A—C4—H4B	109.5
Ni1—O4—H1O4	106.8 (16)	C3—C4—H4C	109.5
Ni1—O4—H2O4	110.0 (15)	H4A—C4—H4C	109.5
H1O4—O4—H2O4	107 (2)	H4B—C4—H4C	109.5
C3—N1—O3	113.48 (13)		
			
N1^i^—Ni1—O1—C1	−178.29 (14)	Ni1—O1—C1—O2	172.23 (11)
O4^i^—Ni1—O1—C1	−87.93 (13)	Ni1—O1—C1—C2	−9.7 (2)
O4—Ni1—O1—C1	92.07 (13)	O2—C1—C2—C3	−162.17 (15)
O1^i^—Ni1—N1—C3	176.44 (15)	O1—C1—C2—C3	19.6 (2)
O1—Ni1—N1—C3	−3.56 (15)	O3—N1—C3—C4	1.8 (2)
O4^i^—Ni1—N1—C3	87.23 (15)	Ni1—N1—C3—C4	−168.07 (13)
O4—Ni1—N1—C3	−92.77 (15)	O3—N1—C3—C2	−177.11 (14)
O1^i^—Ni1—N1—O3	6.79 (10)	Ni1—N1—C3—C2	13.1 (2)
O1—Ni1—N1—O3	−173.21 (10)	C1—C2—C3—N1	−21.1 (2)
O4^i^—Ni1—N1—O3	−82.43 (10)	C1—C2—C3—C4	159.90 (16)
O4—Ni1—N1—O3	97.57 (10)		

Symmetry codes: (i) −*x*, −*y*, −*z*.

### Hydrogen-bond geometry (Å, °)

**Table:**

*D*—H···*A*	*D*—H	H···*A*	*D*···*A*	*D*—H···*A*
O4—H2O4···O2^ii^	0.79 (2)	1.94 (2)	2.7293 (17)	175 (2)
O3—H1O3···O1^i^	0.72 (2)	2.10 (2)	2.7404 (17)	148 (2)
O4—H1O4···O2^iii^	0.87 (3)	1.90 (3)	2.7576 (16)	167 (2)

Symmetry codes: (ii) *x*, *y*−1, *z*; (i) −*x*, −*y*, −*z*; (iii) −*x*−1/2, *y*−1/2, −*z*−1/2.

## References

[bb2] Burla, M. C., Caliandro, R., Camalli, M., Carrozzini, B., Cascarano, G. L., De Caro, L., Giacovazzo, C., Polidori, G. & Spagna, R. (2005). *J. Appl. Cryst.* **38**, 381–388.

[bb3] Duda, A. M., Karaczyn, A., Kozłowski, H., Fritsky, I. O., Głowiak, T., Prisyazhnaya, E. V., Sliva, T. Yu. & Świątek-Kozłowska, J. (1997). *J. Chem. Soc. Dalton Trans.* pp. 3853–3859.

[bb4] Farrugia, L. J. (1997). *J. Appl. Cryst.* **30**, 565.

[bb5] Fritsky, I. O., Kozłowski, H., Kanderal, O. M., Haukka, M., Świątek-Kozłowska, J., Gumienna-Kontecka, E. & Meyer, F. (2006). *Chem. Commun* pp. 4125–4127. 10.1039/b608236j 17024270

[bb6] Fritsky, I. O., Kozłowski, H., Sadler, P. J., Yefetova, O. P., Świątek-Kozłowska, J., Kalibabchuk, V. A. & Głowiak, T. (1998). *J. Chem. Soc. Dalton Trans* pp. 3269–3274.

[bb7] Gumienna-Kontecka, E., Berthon, G., Fritsky, I. O., Wieczorek, R., Latajka, Z. & Kozłowski, H. (2000). *J. Chem. Soc. Dalton Trans* pp. 4201–4208.

[bb8] Khromov, N. V. (1950). *Zh. Obshch. Khim.* **20**, 1858–1867.

[bb9] Mokhir, A. A., Gumienna-Kontecka, E., Świątek-Kozłowska, J., Petkova, E. G., Fritsky, I. O., Jerzykiewicz, L., Kapshuk, A. A. & Sliva, T. Yu. (2002). *Inorg. Chim. Acta*, **329**, 113–121.

[bb10] Moroz, Yu. S., Kulon, K., Haukka, M., Gumienna-Kontecka, E., Kozłowski, H., Meyer, F. & Fritsky, I. O. (2008). *Inorg. Chem.* **47**, 5656–5665. 10.1021/ic702375h 18500796

[bb1] Nonius (2000). *COLLECT* Nonius BV, Delft, The Netherlands.

[bb11] Onindo, C. O., Sliva, T. Yu., Kowalik-Jankowska, T., Fritsky, I. O., Buglyo, P., Pettit, L. D., Kozłowski, H. & Kiss, T. (1995). *J. Chem. Soc. Dalton Trans.* pp. 3911–3915.

[bb12] Otwinowski, Z. & Minor, W. (1997). *Methods in Enzymology*, Vol. 276, *Macromolecular Crystallography*, Part A, edited by C. W. Carter Jr & R. M. Sweet, pp. 307–326. New York: Academic Press.

[bb13] Sheldrick, G. M. (2001). *SADABS* University of Göttingen, Germany.

[bb14] Sheldrick, G. M. (2008). *Acta Cryst.* A**64**, 112–122. 10.1107/S0108767307043930 18156677

[bb15] Sliva, T. Yu., Duda, A. M., Głowiak, T., Fritsky, I. O., Amirkhanov, V. M., Mokhir, A. A. & Kozłowski, H. (1997*a*). *J. Chem. Soc. Dalton Trans* pp. 273–276.

[bb16] Sliva, T. Yu., Kowalik-Jankowska, T., Amirkhanov, V. M., Głowiak, T., Onindo, C. O., Fritsky, I. O. & Kozłowski, H. (1997*b*). *J. Inorg. Biochem* **65**, 287–294.

[bb17] Wörl, S., Fritsky, I. O., Hellwinkel, D., Pritzkow, H. & Krämer, R. (2005*a*). *Eur. J. Inorg. Chem* pp. 759–765.

[bb18] Wörl, S., Pritzkow, H., Fritsky, I. O. & Krämer, R. (2005*b*). *Dalton Trans* pp. 27–29. 10.1039/b417053a 15605143

